# *Glossogyne tenuifolia* Attenuates Proliferation and Migration of Vascular Smooth Muscle Cells

**DOI:** 10.3390/molecules25245832

**Published:** 2020-12-10

**Authors:** Chin-Feng Hsuan, Yung-Chuan Lu, I-Ting Tsai, Jer-Yiing Houng, Shih-Wei Wang, Tzu-Hsien Chang, Ya-Ling Chen, Chi-Chang Chang

**Affiliations:** 1 School of Medicine, College of Medicine, I-Shou University, Kaohsiung 82445, Taiwan; calvin.hsuan@msa.hinet.net (C.-F.H.); ed100614@edah.org.tw (I.-T.T.); shihwei8888@gmail.com (S.-W.W.); 2 Division of Cardiology, Department of Internal Medicine, E-Da Hospital/E-Da Dachang Hospital, Kaohsiung 82445, Taiwan; 3 School of Medicine for International Students, College of Medicine, I-Shou University, Kaohsiung 82445, Taiwan; gregory.yclu@msa.hinet.net; 4 Division of Endocrinology and Metabolism, Department of Internal Medicine, E-Da Hospital, Kaohsiung 82445, Taiwan; 5 Department of Emergency, E-Da Hospital, Kaohsiung 82445, Taiwan; 6 Department of Nutrition, I-Shou University, Kaohsiung 82445, Taiwan; jyhoung@isu.edu.tw (J.-Y.H.); andy3560133@gmail.com (T.-H.C.); 7 Department of Chemical Engineering, I-Shou University, Kaohsiung 84001, Taiwan; 8 Division of Allergy, Immunology, and Rheumatology, Department of Internal Medicine, E-Da Hospital, Kaohsiung 82445, Taiwan; 9 Department of Obstetrics & Gynecology, E-Da Hospital/E-Da Dachang Hospital, Kaohsiung 82445, Taiwan; igiolal2011@gmail.com

**Keywords:** *Glossogyne tenuifolia*, migration, platelet-derived growth factor, proliferation, vascular smooth muscle cell

## Abstract

The proliferation and migration of vascular smooth muscle cells (VSMCs) are essential in the pathogenesis of various vascular diseases, such as atherosclerosis and restenosis. Among the mediators of VSMC during atherosclerosis development, platelet-derived growth factor (PDGF)-BB is a potent mitogen for VSMCs and greatly contributes to the intimal accumulation of VSMCs. *Glossogyne tenuifolia* (GT, Xiang-Ru) is a traditional antipyretic and hepatoprotective herb from Penghu Island, Taiwan. This study evaluated the inhibitory effect of GT ethanol extract (GTE) and GT water extract (GTW) on proliferative and migratory activities in PDGF-BB-induced VSMCs. The experimental results demonstrated that GTE significantly inhibited the PDGF-BB-stimulated VSMC proliferation and migration, as shown by MTT, wound healing, and Boyden chamber assays. GTE was found to have a much more potent inhibitory activity than GTW. Based on the Western blot analysis, GTE significantly blocked the PDGF-BB-induced phosphorylation of NF-κB and mitogen-activated protein kinase (MAPK) pathways, including extracellular signal-regulated kinase (ERK), p38, and JNK, in VSMCs. In addition, GTE retarded the PDGF-BB-mediated migration through the suppression of matrix metalloproteinase (MMP)-2 and MMP-9 expression in VSMCs. Three main ingredients of GT—chlorogenic acid, luteolin-7-glucoside, and luteolin—all showed significant anti-proliferative effects on PDGF-BB-induced VSMCs. As a whole, our findings indicated that GTE has the potential to be a therapeutic agent to prevent or treat restenosis or atherosclerosis.

## 1. Introduction

The morbidity and mortality of cardiovascular diseases are increasing globally. Atherosclerosis is a chronic progressive inflammatory disease and one of the main pathological bases of cardiovascular and cerebrovascular diseases [1]. It is a blood vessel disorder due to arteries thickening from the accumulation of cholesterol, lipids, vascular smooth muscle cells (VSMCs), and immune cells [2,3]. Once the vascular wall is stimulated, low-density lipoprotein (LDL)-C permeates into the sub-endothelial space and oxidizes there, an inflammatory response is subsequently triggered. Monocytes are recruited to the sub-endothelial space and transformed into macrophages. Cytokines and growth factors secreted by macrophages induce the migration and proliferation of VSMCs to the sub-endothelial space. The VSMCs produce an extracellular matrix (ECM), expressing adhesion molecules and secreting cytokines to recruit other inflammatory cells to the sub-endothelial space. They also uptake oxidized LDL-C and give rise to a significant number of lipid-laden cells. These events take part in the formation of atherosclerotic plaque [4]. In this regard, if the proliferation and migration of VSMCs can be suppressed, which can alleviate the thickening of vessel wall, the further development of atherosclerosis would be delayed [5].

The mediators of VSMC during atherosclerosis development include platelet-derived growth factor (PDGF)-BB, thrombin, endothelin-1, IFN-γ and IL-1, which are secreted by platelets, endothelial cells, and leukocytes within the plaque [6]. These mediators stimulate VSMCs to migrate from the vessel media to the intima and secrete ECM components there [7,8]. Among the mediators, PDGF-BB is the most potent VSMC mitogen [9].

Arterial pathogenesis signal cascades can be triggered by PDGF-BB, where the binding of PDGF to its receptor activates its downstream signaling proteins, such as an increasing phosphorylated p38 mitogen-activated protein kinase (MAPK) and activated extracellular signal-regulated kinase (ERK)1/2 [10,11]. The MAPK signaling pathway also plays a crucial role in the regulation of the proliferation, migration, and survival of mammalian cells [12,13]. Accordingly, the experimental model of VSMCs stimulated by PDGF has been widely applied to investigating therapeutic strategies for the treatment or prevention of atherosclerosis [5,14,15,16].

With respect to VSMC migration, the process of the proteolytic degradation or remodeling of the cell-matrix and cell-cell interactions is indispensable, while extracellular proteases, in particular matrix metalloproteinases (MMPs), play essential regulatory roles in the activity. The MMPs are a subgroup of zinc-dependent endopeptidases with overlapping activities against a variety of ECM components including interstitial collagenases (e.g., MMP-1, -8, and -13), gelatinases (MMP-2 and -9), and basement membrane-degrading MMPs. MMP-2 (gelatinase A) and MMP-9 (gelatinase B) are the most important MMPs for degrading type IV collagen, which is the main constituent of the basement membrane [17,18].

*Glossogyne tenuifolia* (GT, Xiang-Ru) is a traditional antipyretic and hepatoprotective herb from Penghu Island of Taiwan. GT has the effects of dispelling wind, clearing away heat, detoxifying, eliminating dampness and swelling, promoting blood circulation, and removing blood stasis [19]. Previous studies have demonstrated that the ethanol extract of GT (GTE) has antioxidant [20,21], anti-cancer [20], antiviral [22], anti-osteoclastogenic [23], and immunomodulation effects [24]. GT was also demonstrated to have the potential to prevent the oxidation of LDL [21] and to protect against the endothelial injury by suppressing the formation of free reactive oxygen species (ROS) [25]. In addition, GTE could attenuate inflammatory mediator synthesis through the NF-κB pathway [26].

Our laboratory confirmed that the GTE can reduce the expression of the adhesion molecules ICAM-1 and VCAM-1 in human umbilical vein endothelial cells (HUVECs), and it can inhibit the adhesion of monocyte cells onto HUVEC cells [27]. Thus, GTE has the potential to prevent atherosclerosis. In the present study, the inhibitory effects of GTE and GT water extract (GTW) on proliferation and migration in PDGF-BB-induced VSMCs were evaluated, and the potential involved mechanisms were also examined.

## 2. Results

### 2.1. Inhibitory Effects of GT Extracts on Proliferation of PDGF-BB-Stimulated VSMCs

**Figure 1:**
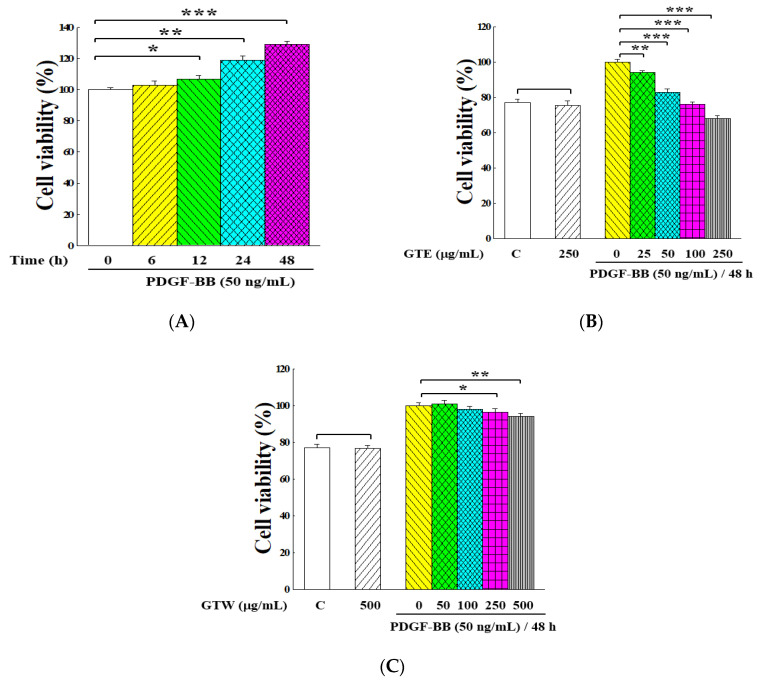
Inhibitory effects of *Glossogyne tenuifolia* ethanol extract (GTE) and *Glossogyne tenuifolia* water extract (GTW) on the proliferation of platelet-derived growth factor (PDGF)-BB-stimulated vascular smooth muscle cells (VSMCs). (**A**) Effect of the treatment time on the proliferation of VSMCs under the stimulation of 50 ng/mL PDGF-BB. (**B**) Effect of the GTE concentration on the proliferation of VSMCs stimulated with 50 ng/mL of PDGF-BB for 48 h. (**C**) Effect of the GTW concentration on the proliferation of VSMCs stimulated with 50 ng/mL of PDGF-BB for 48 h. The cell viability was measured by an MTT assay kit, and the number of cells was regarded as 100% with PDGF-BB stimulation and without sample treatment. Group C was the VSMC growth without PDGF-BB stimulation and GT extract treatment. Data were estimated from five replicate experiments. Significant differences compared with the vehicle (group 0) are denoted as * *p* < 0.05, ** *p* < 0.01, and *** *p* < 0.001.

The effect of GT extracts on the PDGF-BB-induced proliferation of VSMCs was examined. Figure 1A shows that the PDGF-BB-stimulated VSMCs grew rapidly with the elongation of culture time. After 48 h of cultivation, around 25% cells increased. The inhibitory effects of the GTE and GTW extracts on unstimulated and PDGF-BB-stimulated proliferation of VSMCs are indicated in Figure 1B,C. Though both of these two samples exhibited suppression effects on VSMC proliferation in a dose-dependent manner within the tested range, GTE had a higher inhibitory activity than GTW on PDGF-BB-induced VSMC proliferation. Furthermore, the viabilities of unstimulated cells under the treatments of GTE and GTW at their highest doses (250 and 500 μg/mL, respectively) were comparable to those of the control, implying that these two extracts presented an insignificant effect on the growth of unstimulated VSMCs (*p* > 0.05).

### 2.2. Inhibitory Effects of GT Extracts on Migration of PDGF-BB-Stimulated VSMCs

**Figure 2:**
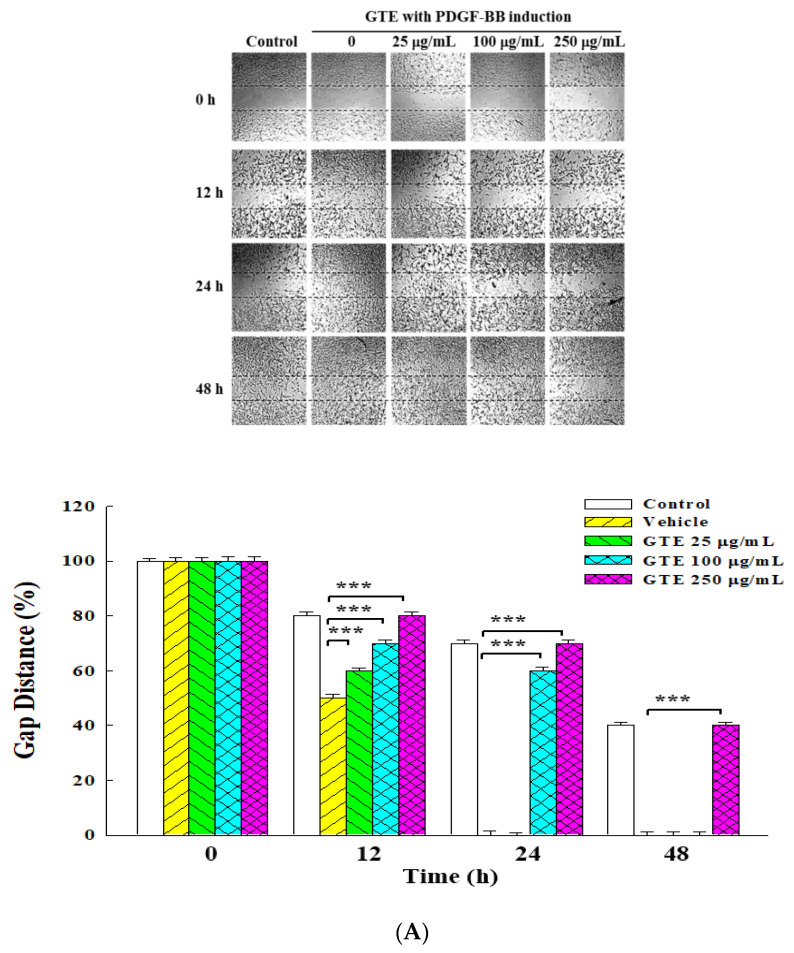

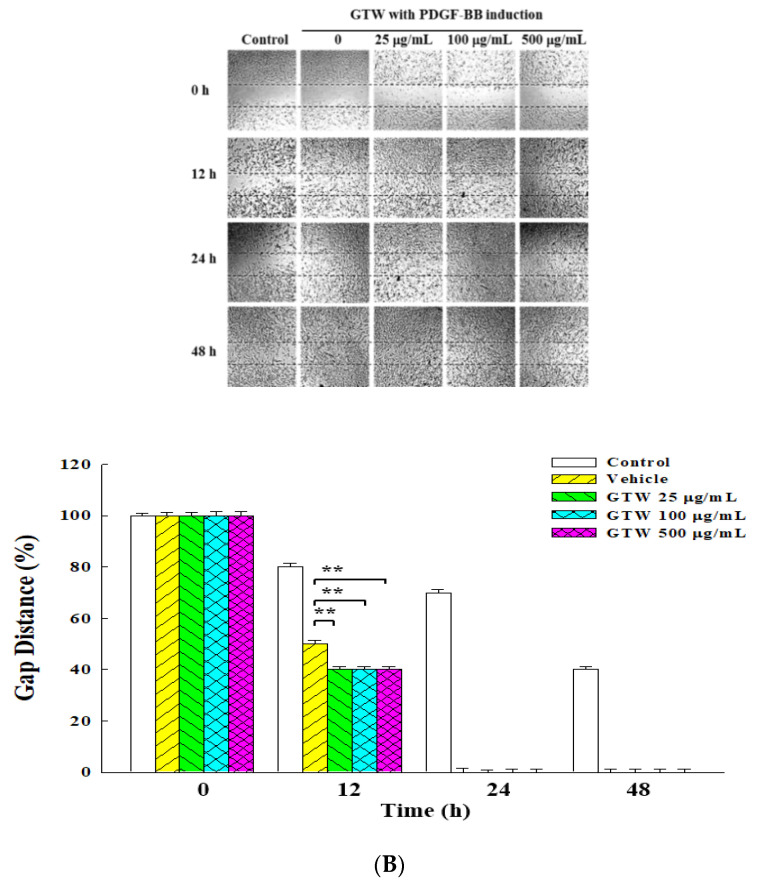
Inhibitory effects of GTE and GTW on the cell motility of PDGF-BB-stimulated VSMCs. Effect of the treatment time at various concentrations of GTE (**A**) and GTW (**B**) on the cell motility under the stimulation with 50 ng/mL of PDGF-BB. The control group was the cell motility of VSMCs without PDGF-BB stimulation. Data were estimated from five replicate experiments. Significant differences compared with the vehicle (group 0) are denoted as ** *p* < 0.01 and *** *p* < 0.001.

A wound healing assay and a Boyden chamber assay were employed to study the anti-migration effect of the two GT extracts on PDGF-BB-stimulated VSMCs. Figure 2 presents the experimental results of the wound healing assay. When not treated with the GT extracts, VSMCs would quickly migrate into the scratched area, and their number rapidly increased with time. When PDGF-BB was added to stimulate the cells, the migration rate increased dramatically. Figure 2A shows that the cells exposed to GTE had a reduced ability to migrate in a concentration-dependent manner. Meanwhile, GTW showed no suppression effect on the migration of PDGF-BB-stimulated VSMCs (Figure 2B).

**Figure 3:**
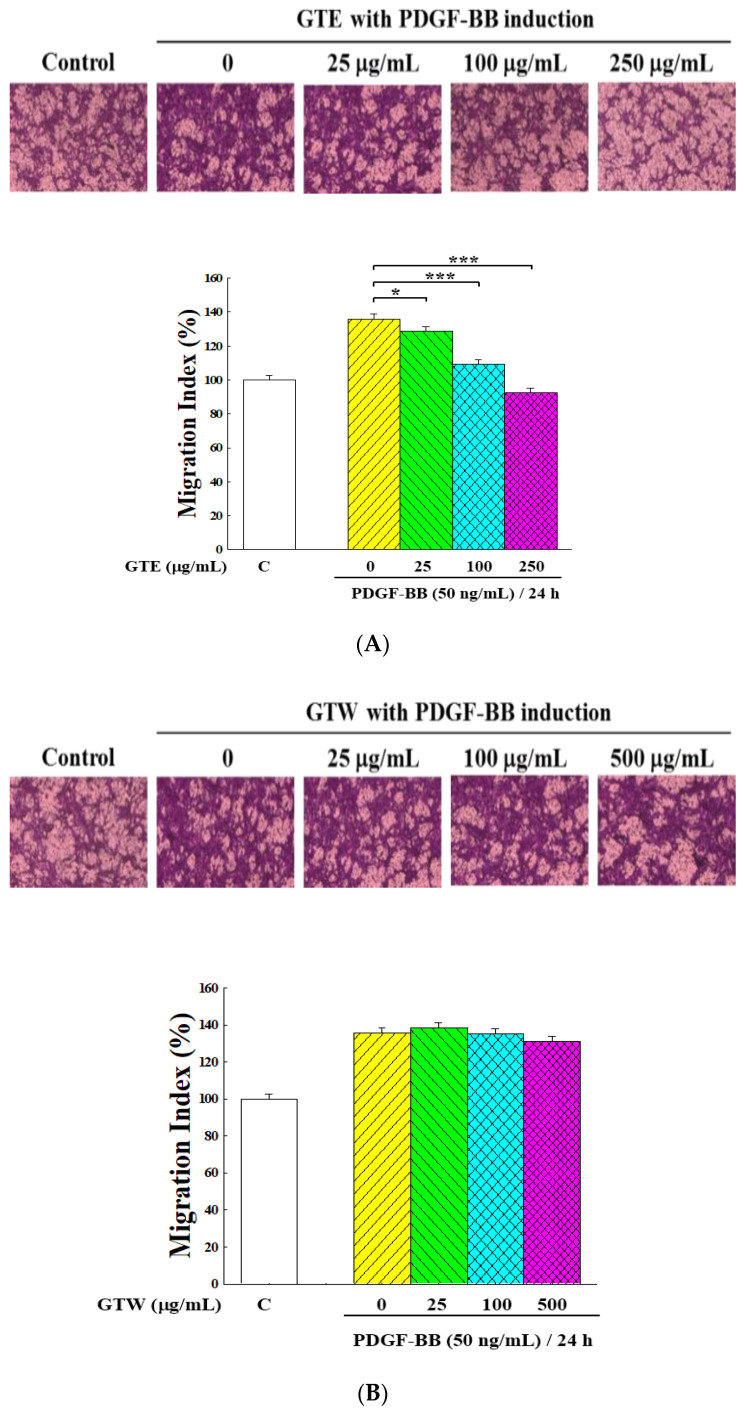
Inhibitory effects of GTE and GTW on the cell migration of PDGF-BB-stimulated VSMCs by a transwell assay. The migration assay was performed using a Boyden chamber. The PDGF-BB-stimulated VSMCs were treated with various concentrations of GTE (**A**) and GTW (**B**) for 24 h. Group C was the VSMC growth without PDGF-BB stimulation and GT extract treatment. Crystal violet was used to stain the cells that penetrated through the polycarbonate membrane to the surface under the filter, photographed with a phase-contrast microscope, and the number of penetrated cells were counted. Data were obtained from five independent experiments. Significant differences compared with the vehicle (group 0) are denoted as * *p* < 0.05 and *** *p* < 0.001.

A cell migration assay with the Boyden chamber was used to further examine the effect of GT extracts on the motility of VSMCs. As shown in Figure 3, the number of cells that migrated to the lower chamber remarkably increased with the stimulation of PDGF-BB. Figure 3A demonstrates that GTE treatment dramatically suppressed the PDGF-BB-induced migration of VSMCs in a dose-dependent manner when compared to that of vehicle (GTE concentration = 0 μg/mL, *p* < 0.001). On the other hand, GTW only exhibited a little inhibitory effect (Figure 3B).

As only GTE displayed a strong inhibitory effect on PDGF-BB-induced proliferation and migration, this study applied GTE in the subsequent experiments.

### 2.3. GTE Suppresses NF-κB and MAPK Signaling Pathways in PDGF-BB-Stimulated VSMCs

**Figure 4:**
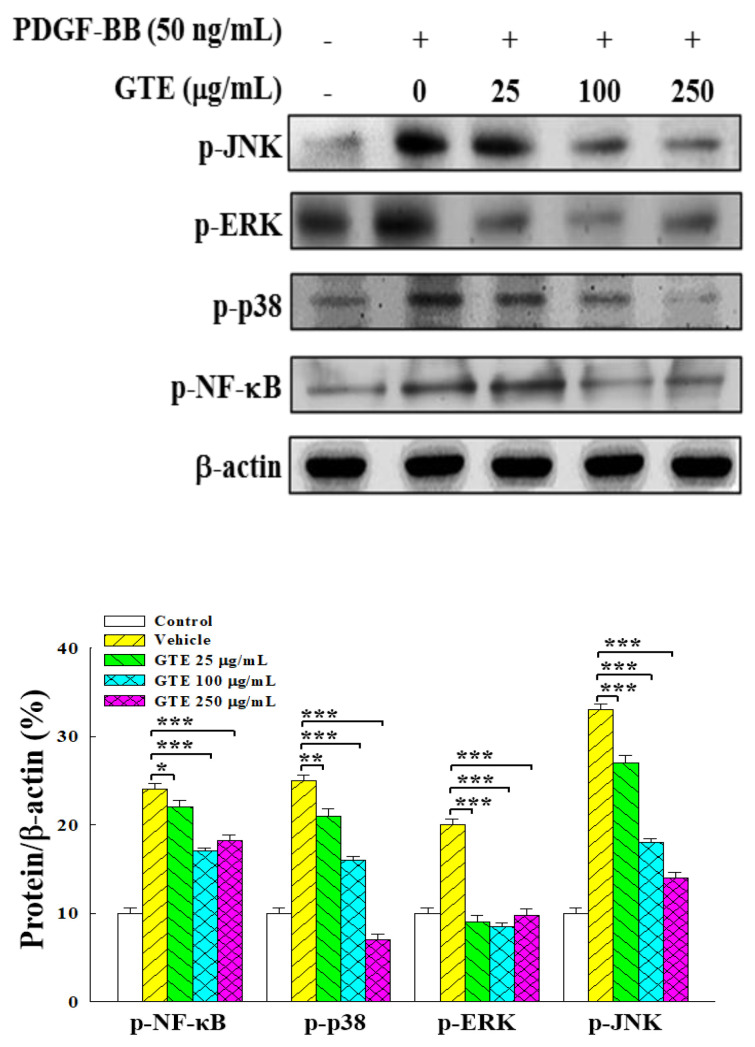
Effects of GTE on the PDGF-BB-stimulated activation of downstream signaling pathways in VSMCs. PDGF-BB-stimulated VSMCs were treated with 0–250 μg/mL of GTE for 48 h. The cell extracts were subjected to electrophoresis on SDS-polyacrylamide gels with a subsequent enzyme immunoassay using enhanced chemiluminescence (ECL). β-Actin was used as internal control. Densitometric analyses were conducted and normalized to the corresponding total protein. Data were estimated from three independent experiments. Significant differences compared with the vehicle (group 0) are denoted as * *p* < 0.05, ** *p* < 0.01, and *** *p* < 0.001.

The NF-κB and MAPK signaling pathways are involved in regulating the function of VSMCs and have been suggested as the key signal cascades in the formation of atherosclerosis [28,29]. The effect of GTE treatment on the NF-κB and MAPK signaling pathways was examined by Western blot analysis. Figure 4 shows that PDGF-BB significantly increased the phosphorylation of NF-κB, p38 MAPK (p38), ERK, and JNK in PDGF-BB-induced VSMCs. The treatment of GTE reduced the phosphorylation of these four proteins in a dose-dependent manner, suggesting that GTE could substantially attenuate the NF-κB and MAPK signaling pathways in VSMCs.

### 2.4. GTE Inhibits VSMC Migration by Suppressing the Expression of MMP-2 and MMP-9

**Figure 5:**
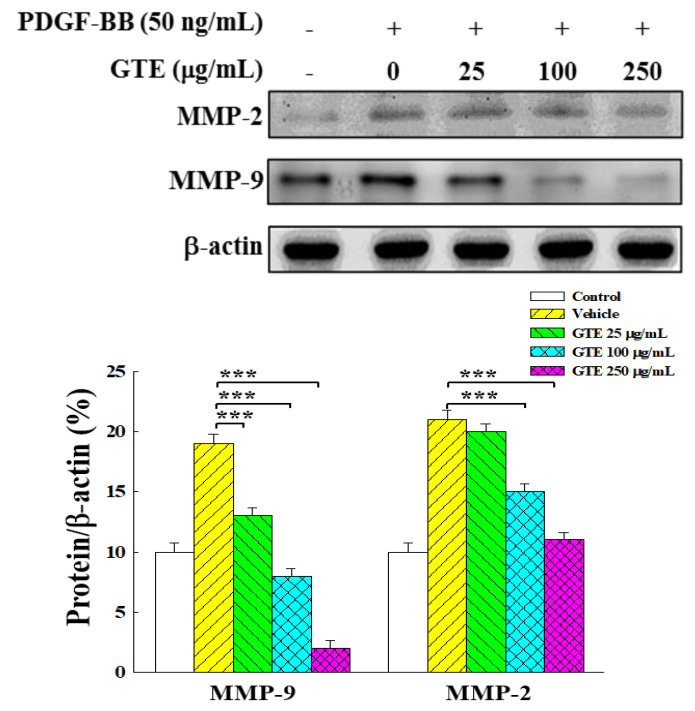
Effects of GTE on matrix metalloproteinase (MMP)-2 and MMP-9 expressions in PDGF-BB-stimulated VSMCs. Expressions of MMP-2 and MMP-9 were analyzed by Western blotting. PDGF-BB-stimulated VSMCs were treated with 0–250 μg/mL of GTE for 48 h. Data were estimated from triplicate experiments. A significant difference compared with the vehicle (group 0) is denoted as *** *p* < 0.001.

MMPs are involved in the regulation of a variety of processes associated with vascular structure and remodeling, VSMC migration, and the development of atherosclerosis [18]. The Western blot analysis revealed that PDGF-BB markedly increased the expression levels of MMP-2 and MMP-9 (Figure 5). The treatment of GTE could dramatically decrease the expression of MMP-2 and MMP-9 dose-dependently, indicating that GTE could suppress the expression of MMP-2 and MMP-9 in PDGF-BB-stimulated VSMCs.

### 2.5. Inhibitory Effects of Main Ingredients on Proliferation of PDGF-BB-Stimulated VSMCs

**Figure 6:**
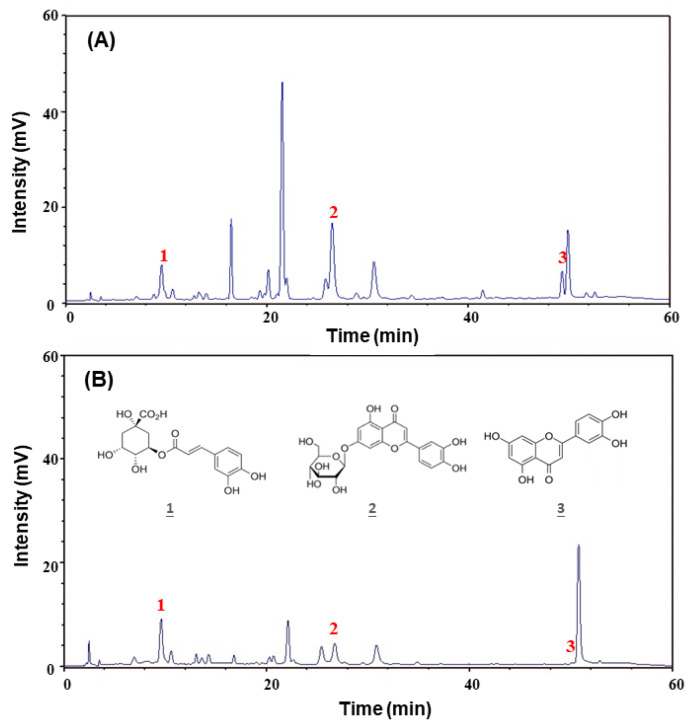
HPLC chromatogram of the GTE (**A**) and GTW (**B**). Identified components: 1: chlorogenic acid; 2: luteolin-7-glucoside; and 3: luteolin.

The main bioactive ingredients of the GT extracts are polyphenol and flavonoid compounds [30]. Figure 6 shows the HPLC chromatograms of the two extracts. Three components were identified as chlorogenic acid (CGA—peak 1 with a retention time (RT) = 9.6 min), luteolin-7-glucoside (lut-7-g—peak 2 with an RT = 25.4 min), and luteolin (lut—peak 3 with an RT = 50.1 min). Table 1 demonstrates that the GTE extract contained higher amounts of lut-7-g, lut, total polyphenols, and total flavonoids than the GTW extract.

**Figure 7:**
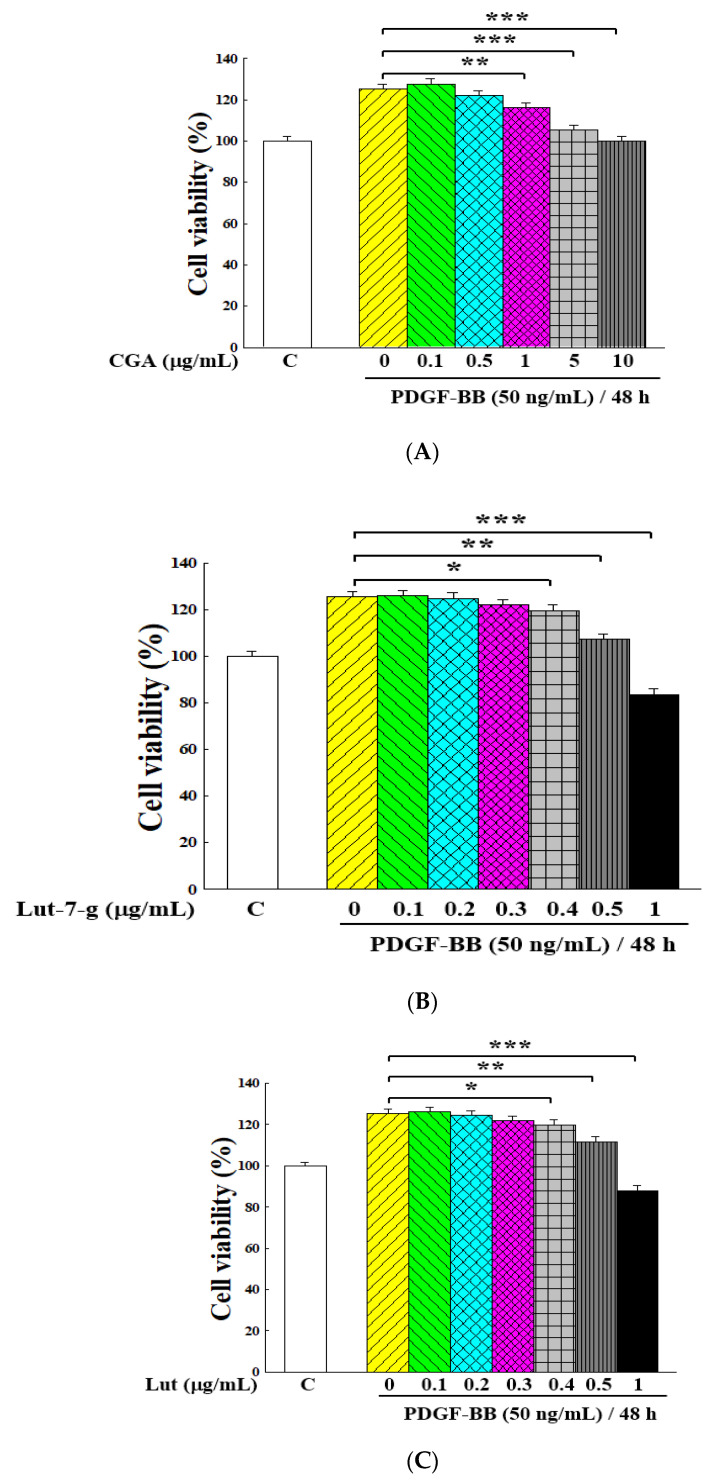
Inhibitory effects of the main GT ingredients on the proliferation of PDGF-BB-stimulated VSMCs: CGA (**A**), lut-7-g (**B**), and lut (**C**). The cell proliferation was conducted under the stimulation of 50 ng/mL of PDGF-BB for 48 h. The cell viability was measured by an MTT assay kit, and the number of cells was regarded as 100% with PDGF-BB stimulation and without sample treatment. Group C was the VSMC growth without PDGF-BB stimulation and sample treatment. Data were estimated from five replicate experiments. Significant differences compared with the vehicle (group 0) are denoted as * *p* < 0.05, ** *p* < 0.01, and *** *p* < 0.001.

Figure 7 demonstrates the inhibitory effects of these three ingredients on the proliferation of PDGF-BB-induced VSMCs. For CGA, it had a significant effect at above 1 μg/mL (*p* < 0.01, Figure 7A). For lut-7-g and lut, the inhibitory effect was significant at above 0.4 μg/mL (*p* < 0.05, Figure 7B,C). This illustrates that these three components could effectively inhibit the proliferation of PDGF-BB-stimulated VSMCs.

## 3. Discussion

The abnormal proliferation and migration of VSMCs contribute to the progression of the vascular pathophysiological process of atherosclerosis and restenosis [31,32]. The suppression of abnormal VSMC proliferation and migration has been regarded as a feasible strategy against vascular restenosis or neointima formation in atherosclerosis [15,16,33]. PDGF is a potent inducer of the VSMC proliferation and migration [13,14,34]. The experimental results of this study revealed that GTE can remarkably suppress the PDGF-BB-induced cell growth and mobility of VSMCs.

Many studies have reported that the proliferation and mitogenic stimulus within VSMCs are translated by some specific transduction signal cascades, including NF-κB, MAPK, and MMP pathways, and they are involved in the regulation of atherosclerosis [13,18,29,33]. The transcription factor NF-κB is generally involved in inflammatory and immune responses, and it regulates cell cycle progression and proliferation in many different types of cells [35]. Activated nuclear NF-κB has been found in macrophages, endothelial cells, and VSMCs in the intima and media of atherosclerotic vessel [31,36,37]. Mehrhof et al. indicated that essential pathogenetic functions of NF-κB in the development of atherosclerosis involve the signaling of cellular proliferation, apoptosis, and inflammation of VSMCs [37]. Therefore, NF-κB plays a crucial role in the development of atherosclerosis. This study shows that GTE suppressed the phosphorylation of NF-κB in PDGF-BB-stimulated VSMCs, implying that GTE could inhibit the cell growth of PDGF-BB-stimulated VSMCs through the NF-κB pathway and attenuate the development of atherosclerosis.

MAP kinases (MAPKs), including ERK, p38, and JNK, regulate a variety of cellular programs by relaying extracellular signals to intracellular responses in many cell types. Studies have suggested that MAPK signaling is an important pathway in the PDGF-mediated proliferation and migration of VSMCs [13,38,39]. Though the importance of ERK and p38 in PDGF-induced VSMC responses has been addressed, only a few studies have reported the impact of JNK [14,15,33,34]. This study demonstrated that GTE could attenuate the PDGF-BB-induced phosphorylation of ERK, p38, and JNK, thus indicating that GTE exerts an inhibitory effect on the proliferation and migration of VSMCs via this pathway. The experimental results of Figure 4 show that when GTE concentration was increased to 250 μg/mL, the expression of p-NF-κB and p-ERK did not decrease but, in fact, increased. Regarding this point, it is possible that when the concentration of GTE increased to 250 μg/mL, some of its ingredients started to show more obvious effects on the phosphorylation of NF-κB and ERK, leading to the increase of the expression of p-NF-κB and p-ERK. In this regard, further study is needed to investigate what these ingredients are and how they influence the phosphorylation of NF-κB and ERK.

Several enzymes that degrade the basement membrane, such as MMPs, are assumed to play an important role in the migration of VSMCs. MMPs may degrade or remodel the ECM and remove the basement membrane around VSMCs. In particular, activated forms of MMP-2 and MMP-9 have been identified as important roles in VSMC migration due to their ability to cleave type IV collagen [5,15,18]. Our results showed that the treatment with GTE significantly down-regulated the PDGF-BB-induced MMP-2 and MMP-9 expression in a dose-dependent manner, suggesting that the anti-migration effect of GTE was associated with inhibition of enzymatic degradation processes of MMPs.

Some natural compounds have been verified to have inhibitory effects on the abnormal proliferation and migration of VSMCs, including polyphenols, flavonoids, terpenes, and alkaloids [40]. In the treatment of cardiovascular diseases, research on flavonoids and polyphenols has mainly focused on reducing the risk of hypertension, atherosclerosis, vascular inflammation, and oxidative stress, as well as the impact on related signaling pathways [40,41,42]. The flavonoid compounds of CGA, lut, and lut-7-g have been reported as the main active compounds of GT [20,21,30]. These ingredients have been shown to have antioxidant, anti-inflammatory, antiviral, anti-osteoclastogenic, immunomodulatory, and anti-cancer activities [27]. Our previous study demonstrated that the treatment of GTE, lut, and lut-7-g could inhibit the adhesion of THP-1 to TNF-α-activated HUVECs and reduce the cell surface expression of adhesion molecules in HUVECs [27].

In inhibiting the abnormal proliferation and migration of VSMCs, CGA was reported to possess inhibitory effects on the proliferation of hypoxia-induced pulmonary artery smooth muscle cells via c-Src and the Shc/Grb2/ERK2 signaling pathway, as well as on monocrotaline-induced pulmonary arterial hypertension in rats through vascular remodeling [43]. Lut-7-g was demonstrated to have the inhibitory activity on the PDGF-BB-induced proliferation of VSMCs via the blocking of phospholipase C (PLC)-γ1, Akt, and ERK1/2 phosphorylation [44]. Luteolin could inhibit hydrogen peroxide-induced VSMCs proliferation and migration by suppressing the Src and Akt signaling pathways [45]. Luteolin could alleviate the cell migration and proliferation of angiotensin II-stimulated VSMCs by attenuating the formation of VEGF, Nox4, and p-Akt in endothelial cells [46] by reducing angiotensin II-stimulated ROS production and decreasing ERK1/2, p-ERK1/2, p-p38, MMP-2 levels in VSMCs [47]. Su et al. found that a luteolin treatment could significantly decrease the blood pressure and media thickness of the aorta in spontaneous hypertensive rats [48].

In the solvent extraction process, flavonoids and polyphenols are more suitable for extraction with ethanol than water. Therefore, in the present study, GTE contained higher amounts of flavonoids and polyphenols than GTW. This may explain why GTE had a more potent inhibitory effect than GTW on the growth and migration of VSMCs. In addition, the use of ethanol as a solvent can generally extract more components than that of water extraction, possibly leading to a higher inhibitory activity of GTE on cell proliferation and migration. Further studies are needed to examine the differences in the activity between GTE and GTW.

The results of Figure 1 show that GTE inhibited cell growth by around 30% at a concentration of 250 μg/mL, with its lut-7-g and lut contents of 11.5 and 0.93 μg/mL, respectively. Figure 7 shows that both lut-7-g and lut could inhibit cell growth by around 35–40% at a concentration of 1 μg/mL. This difference indicates that the inhibitory activity of GTE on cell proliferation cannot be simply expressed by the content of lut-7-g and lut. Our previous study reported that both lut-7-g and lut had significant anti-proliferation effects on Hep G2 liver cancer cells [20], in which the IC_50_ values of lut-7-g and lut were 14.81 and 8.25 μg/mL, respectively. However, for GTE, when the treatment concentration was 250 μg/mL, it could only inhibit the growth of cancer cells by about 25%, which was similar to the present study. Therefore, there should be other substances in GTE that would offset the inhibitory ability of lut-7-g and lut, and such activity would thus require further exploration.

The main reason for using extracts in this study, instead of pure compounds, was to evaluate the potential of GT extracts as adjuvant therapeutic agents. The difference from the study of pure compounds is that an extract is a mixture and may contain different components that may exhibit interaction effects and promote or offset the activity of one another. From the above discussion, some discrepancies between the extract and the bioactive compound were observed. The results imply some other components of GTE may have more obvious interactions, and this would be worthy of further investigation.

## 4. Materials and Methods

### 4.1. Preparation of GT Extracts

The GT plant was bought from Qian-Yi Tang herb store in Magong City, Penghu Island, Taiwan. The GT was collected in the countryside surrounding Huxi Township (latitude, 23°60′ N; longitude, 119°65′ E). The species of GT was confirmed by its 5.8 s DNA sequence, and its fidelity was near 100% when compared with the data in NCBI’s DNA database (Accession number: AY429090) [20]. The raw material was deposited to be No. ISU-JYH-001 in the Herbarium of I-Shou University (Kaohsiung, Taiwan). During the preparation of GTE, 1 kg of dry whole plant materials of GT was crushed and extracted by 8 L of 75% ethanol for one day. The extraction was carried out for three times. All the extracted solutions were poured together and filtrated by filtration paper. The filtrates were collected and concentrated with a vacuum evaporator (Panchum Scientific Co., Kaohsiung, Taiwan). The extract samples were dried with a freeze-dryer (Panchum Scientific Co.). The extraction yield was 9.78%.

While preparing GTW, 1 kg of crushed GT powder was extracted by 8 L of hot distilled water for 3 h. The extraction was conducted three times. The extracted solution was filtrated, concentrated, and freeze-dried. The extraction yield was 7.64%.

### 4.2. Cell Culture

The rat aortic VSMC line (No. R6110) was purchased from ScienCell Research Laboratories (Carlsbad, CA, USA). The cultivation of the cell line was grown in a Smooth Muscle Cell Medium (SMCM; ScienCell Research Laboratories). The cells were cultivated at 37 °C with 5% CO_2_ and 95% air in a humidified incubator.

VSMCs were cultured in 12-well plates with 2 × 10^4^ cells, and the indicated concentration of extract or main ingredient of GT with or without PDGF-BB (Sigma-Aldrich, St. Louis, MO, USA) was added. The solvents of GTE and GTW samples were dimethyl sulfoxide (DMSO) and water, respectively. The final solvent concentration in the medium was less than 0.1%, and the cell growth would not have been affected by the solvent below this concentration. After incubation for the specified time, the culture medium was removed, 100 μL of the culture medium containing 0.5 mg/mL of an MTT assay kit (Sigma-Aldrich) was added, and the mixture then incubated for another 4 h. Subsequently, the medium solution was withdrawn and replaced with 100 μL of DMSO, and the plate was shaken until the crystal was completely dissolved. The proliferation rate of VSMCs was measured at 570 nm using an ELISA reader (Model 550, Bio-Rad Laboratories, Hercules, CA, USA).

### 4.3. Cell Migration by Wound Healing Assay

An aliquot of 1 × 10^6^ VSMCs was cultured for 24 h with a culture insert (Sigma-Aldrich). After the cultivation, the insert was removed, and the culture medium containing certain dose of the GT extract with 50 ng/mL PDGF-BB was added to induce a migrating zone. The cells were allowed to migrate for 48 h, and the variations of gap distances were measured accordingly. The migrated cells were photographed by an inverted phase-contrast microscope (Nikon Eclipse TS100, Chiyoda-ku, Tokyo, Japan).

### 4.4. Cell Migration Assay by Boyden Chamber

The CytoSelect™ 24-well cell migration assay (Cell Biolabs, San Diego, CA, USA) was applied to evaluate the PDGF-BB-mediated VSMC migration. To determine the effects of extract samples on cell migration, 1 × 10^6^ VSMCs with 50 ng/mL of PDGF-BB was placed with different concentrations of the GT extract in 100 μL of a serum-free medium on an inserted polycarbonate membrane, and then it was put into the bottom chamber that contained SMCM with 10% FBS as a chemo-attractant. After incubating for 24 h, the non-migrated cells in the upper chamber were cleared. The invaded cells were cleaned with a phosphate buffer solution (PBS—0.01 M and pH 7.2) three times, fixed with methanol, stained with 400 μL of crystal violet, and photographed under a phase-contrast microscope. The numbers of invaded cells were counted.

### 4.5. Western Blot Analysis

The cell lysis was conducted with the method described in our previous paper [49]. The concentration of harvested protein was determined using a Bradford Protein Assay Kit (Bio-Rad, California, CA, USA). Samples with 40 μg of denatured proteins were resolved on 10% sodium dodecyl sulfate polyacrylamide gel electrophoresis (SDS-PAGE) and then transferred onto a polyvinylidene difluoride (PVDF) membrane (Bio-Rad) that was blocked with 5% nonfat milk in Tris-buffered saline at 4 °C for 1 h. The indicated primary antibody at a 1:5000 dilution was used and then hybridized with horseradish peroxidase-conjugated secondary antibodies at a 1:2000 dilution. The used antibodies were all purchased from Sigma-Aldrich, and their identical numbers are listed in Table 2. Enhanced chemiluminescence (ECL) and Western blotting detection reagents (Amersham Bioscience, Uppsala, Sweden) were adopted to detect the protein levels. The densitometric analyses were conducted by ChemiDoc XRS+ System (Bio-Rad).

### 4.6. Determination of the Main Ingredients Amounts by HPLC

The amounts of main ingredients of GT were determined by HPLC (Model L-7100, Hitachi, Tokyo, Japan). Each extract was dissolved in methanol. The diluted samples were analyzed by an Ascentis^TM^ C18 column (No. 581325-U, 5 μm, 250 × 4.6 mm; Supelco, Bellefonte, PA, USA). The mobile phase was the solution of methanol/0.05% acetic acid in water (40:60, *v*/*v*). The flow rate was 1.0 mL/min. The concentration of the extract sample was 1000 μg/mL. The detection was conducted at 350 nm. The compounds used to build the calibration curves and applied in the cytotoxicity experiment on VSMCs were purchased from Sigma-Aldrich.

### 4.7. Determination of Total Polyphenols Content and Total Flavonoids Content

The total amount of phenolic and flavonoid compounds of each extract was determined according to the methods of Tsai et al. [29]. For the determination of total polyphenols content, an aliquot of 0.15 mL extract sample was mixed with 0.75 mL of a 0.2 N Folin-Ciocalteu reagent (Sigma-Aldrich) and 0.6 mL of a 7.5% sodium carbonate solution. The mixture was kept at room temperature for 30 min and then measured at 765 nm with a spectrophotometer (Ultrospec 2100 pro, GE Health-care, Amersham Place, UK). The total polyphenols content was estimated from the calibration curve that was built using gallic acid as a standard and is expressed as gallic acid equivalents of dry extract.

To determine the total flavonoids content, an aliquot of 0.3 mL of the sample, 1.2 mL of deionized water, and 0.075 mL of 15% Na_2_CO_3_ was added to a test tube. After 5 min, 0.15 mL of 10% AlCl_3_ was added and kept at room temperature for 6 min. A solution containing 0.5 mL of 1 M NaOH and 0.275 mL of deionized water were added. After mixing, the absorbance of the solution was measured at 510 nm. The total flavonoids content was estimated from the calibration curve built using catechin as a standard and is expressed as catechin equivalents of dry extract.

### 4.8. Statistical Analysis

All experiments were conducted for three-to-five independent replicates. The data are expressed in terms of mean and standard deviation. Student’s *t*-test was applied to analyze the statistical differences (* *p* < 0.05, ** *p* < 0.01 and *** *p* < 0.001). Microsoft Excel software (Office 2019, Microsoft Software Inc., Redmond, WA, USA) was used to calculate and analyze experimental data.

## 5. Conclusions

This study demonstrated that GTE has a potent inhibitory effect on the proliferative and migratory activities of PDGF-BB-stimulated VSMCs. Based on Western blot analysis, GTE could not only attenuate the phosphorylation of NF-κB and MAPK, including the ERK, p38, and JNK pathways in VSMCs, but also alleviate the PDGF-BB-mediated migration through the suppression of MMP-2 and MMP-9 expression in VSMCs. Additionally, the three major ingredients of GT were shown to have significant inhibitory effects on the proliferation of PDGF-BB-stimulated VSMCs. Therefore, GTE has a potential to be a therapeutic agent to prevent or treat restenosis or atherosclerosis.

## Figures and Tables

**Table 1:** Main ingredients analysis of the GT extracts.

Ingredient	Concentration (mg/g Extract)	
	GTE	GTW
Chlorogenic acid (**1**)	9.03 ± 0.25	9.45 ± 0.34
Luteolin-7-glucoside (**2**)	46.23 ± 1.42	15.40 ± 0.85
Luteolin (**3**)	3.71 ± 0.11	1.70 ± 0.06
Total polyphenols content	64.04 ± 1.68	26.15 ± 0.96
Total flavonoids content	52.97 ± 1.14	17.49 ± 0.67

**Table 2:** Company and their corresponding identical numbers of the used antibodies.

Antibody	Company	Identical Number
p-JNK	Sigma-Aldrich	SAB4504450
p-ERK	Sigma-Aldrich	SAB4301578
p-p38	Sigma-Aldrich	SAB4301534
p-NF-κB	Sigma-Aldrich	SAB4502609
MMP-2	Sigma-Aldrich	SAB5700824
MMP-9	Sigma-Aldrich	SAB5700152
β-Actin	Sigma-Aldrich	SAB3500350
